# Predicting chromosome 1p/19q codeletion by RNA expression profile: a comparison of current prediction models

**DOI:** 10.18632/aging.101795

**Published:** 2019-02-02

**Authors:** Zhi-liang Wang, Zheng Zhao, Zheng Wang, Chuan-bao Zhang, Tao Jiang

**Affiliations:** ^1^Beijing Neurosurgical Institute, Capital Medical University, Beijing, China; ^2^Department of Neurosurgery, Beijing Tiantan Hospital, Capital Medical University, Beijing, China; ^3^China National Clinical Research Center for Neurological Diseases, Beijing, China; ^4^Center of Brain Tumor, Beijing Institute for Brain Disorders, Beijing, China

**Keywords:** oligodendroglioma, 1p/19q codeletion, prediction, smoother, method

## Abstract

Background: Chromosome 1p/19q codeletion is increasingly being recognized as the crucial genetic marker for glioma patients and have been included in WHO classification of glioma in 2016. Fluorescent in situ hybridization, a widely used method in detecting 1p/19q status, has some methodological limitations which might influence the clinical management for doctors. Here, we attempted to explore an RNA sequencing computational method to detect 1p/19q status.

Methods: We included 692 samples with 1p/19q status information from TCGA cohort as training set and 222 samples with 1p/19q status information from REMBRANDT cohort as validation set. We reviewed and compared five tools: TSPairs, GSVA, PAM, Caret, smoother, with respect to their accuracy, sensitivity and specificity.

Results: In TCGA cohort, the GSVA method showed the highest accuracy (98.4%) in predicting 1p/19q status (sensitivity=95.5%, specificity=99.6%) and smoother method showed the second-highest accuracy (accuracy=97.8%, sensitivity=96.4%, specificity=98.3%). While in REMBRANDT cohort, smoother method exhibited the highest accuracy (98.6%) (sensitivity= 96.7%, specificity=98.9%) in 1p/19q status prediction.

Conclusions: Our independent assessment of five tools revealed that smoother method was selected as the most stable and accurate method in predicting 1p/19q status. This method could be regarded as a potential alternative method for clinical practice in future.

## INTRODUCTION

Glioma is the most common and deadliest malignant primary brain tumor in adults [1]. Oligodendroglial tumors, including oligodendrogliomas and oligoastrocytomas, are the second common type of glioma [2–5]. Chromosome 1p/19q codeletion, complete deletion of both the short arm of chromosome 1 and the long arm of chromosome 19, is the specific hallmark of oligodendrogliomas. The frequency of this genetic aberration in oligoastrocytoma and oligodendroglioma are 50%~70% and 70% ~80%, respectively [6].

Nowadays, 1p/19q codeletion is increasingly recognized as a crucial genetic aberration in glioma patients and was first time included in the WHO classification of brain tumor in 2016 [7]. This pathognomonic biomarker is thought to commonly occur in the early phase of glioma development [8]. In addition, numerous studies explored the clinical significance of 1p/19q codeletion and found that it is a strong independent favorable prognosticator of overall survival (OS) and progression free survival (PFS) for glioma patients [8–11], and patients with this aberration would benefit from radiation therapy plus chemotherapy in comparison with radiation therapy alone after surgery [10]. Hence, prediction of 1p/19q status accurate become particularly critical for the precision medical in glioma patients.

Fluorescent in situ hybridization (FISH), targeting 1p36/1p21 and 19q13/19p13 regions via fluorophore-labelled DNA probes [12], was used as standard protocol to detect 1p/19q status in most hospitals [13]. However, FISH has some methodological limitations neuropathologists need to be aware of in clinical practice. Firstly, probes designed for chromosome 1p and 19q span a long region that may not identify small interstitial and terminal deletions. Secondly, FISH may not detect hemizygous deletions if there is loss of one allele and reduplication of the other allele [14]. Thirdly, FISH analysis is time-consuming and subjective which requires experienced pathologist to ensure result accuracy [15]. The incorrect 1p/19q status detecting by FISH may cause improper treatment strategy for patients [16]. Moreover, using FISH to detect 1p/19q status exerts a financial burden on glioma patients and fails to get more genetic alterations information.

Nowadays, next generation RNA sequencing (RNA-seq) technologies greatly promote the exploration of the complex and dynamic nature of cancer [17] and could provide insights to previously undetected changes occurring in disease [18]. RNA sequencing data has been successfully applied in identifying single nucleotide variants mutation [19], alternative splicing [20], fusion genes [21] and RNA editing [22]. Comprehensive understanding of the gene expression profile variation caused by copy number variation provide us possibility to detect 1p/19q status.

However, the comprehensive study that integrated RNA-seq data analysis methods to predict 1p/19q status has not been conducted yet. Therefore, in this study, we reviewed and assessed five methods which were designed to detect gene expression variations with RNA-seq data, and attempted to find out a precise, objective and cost-effective method to replace FISH for identifying 1p/19q status in clinical practice in the future.

## RESULTS

### Chromosome 1p/19q co-deleted patients exhibited a distinct expression profile

In order to assess the feasibility of predicting 1p/19q status with RNA expression data, we used whole genome expression profiling (20501 genes) from TCGA dataset to explore the relationship between 1p/19q status and gene expression profiles. As shown in Supplementary Figure 1A, hierarchical cluster method separated the dendrogram into two branches. The first branch was consisted by five subgroups while the second branch contained only one subgroup. The majority of 1p/19q co-deleted patients were in group 1, 3 and 5, while the 1p/19q intact patients were mainly in the rest of groups (group 2, 4, 6). The result indicated that there were some obvious differences in expression profiles between 1p/19q intact patients and 1p/19q co-deleted patients. However, the classification process was interfered by noisy genes and it was hard for us to predict 1p/19q status clearly with raw RNA sequencing data. Then we tried to change the threshold of gene expression to improve the classification accuracy in detecting 1p/19q status. Previously studies used MAD value to evaluate highly variable expression genes with RNA sequencing data [2, 23]. Here, we used highly variable genes (MAD >2, 886 genes) for hierarchical clustering, and the distance between 1p/19q co-deleted samples (Supplementary Figure 1B) was closer than clustering with whole gene expression profiles (Supplementary Figure 1A). And the hierarchical assignment in clustering chromosome 1p and 19q genes (n=1775) expression profile exhibited a similar result (Supplementary Figure 1C). Those results indicated that the significant differences of RNA sequencing data between 1p/19q co-deleted and intact patients could provide a feasibility to predict 1p/19q status by RNA expression data.

### Overview of five methods in predicting 1p/19q status

Based on this finding, we selected five methods which have been used to process RNA sequencing data to identify 1p/19q status.

**Figure 1:**
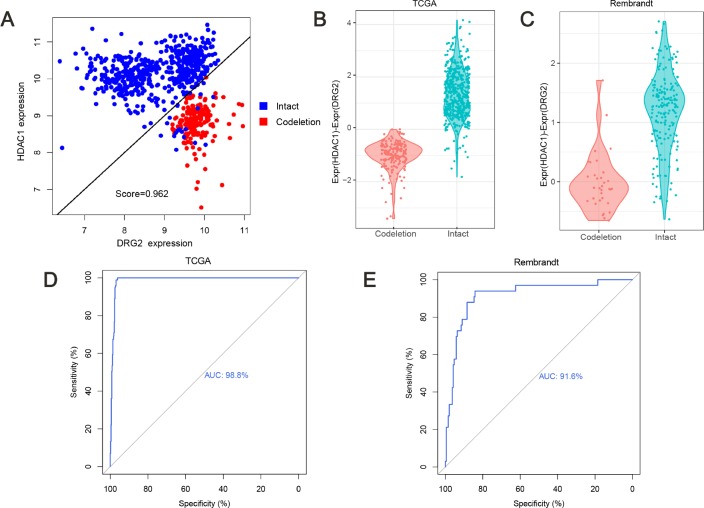
**Predicting 1p/19q status by TSPair algorithm.** (**A**) HDAC1 and DRG2 pair was the top scoring pair in predicting 1p/19q (score=0.962). The expression values of training set were normalized as Expr=log2(RSEM+1). (**B**) the values (HDAC1 - DRG2 expression values) were significantly different (p=0.0063) between 1p/19q co-deleted group and intact group in TCGA cohort. (**C**) the values (HDAC1 - DRG2 expression values) were significantly different (p<0.0001) between 1p/19q co-deleted group and intact group in Rembrandt cohort. (**D** and **E**) ROC curve for 1p/19q status prediction in TCGA cohort and Rembrandt cohort, AUC=0.988 and 0.916, respectively.

TSPairs method which compared the two genes expression ratio was used in breast cancer and lung adenocarcinoma for classification and prognosis [24, 25]. In the training dataset, with the *tspcalc* function, HDAC1(Histone deacetylase 1) and DRG2 (Developmentally regulated GTP binding protein 2) gene pair which had the highest TSP score (0.962) was selected as the most consistent switch (Figure 1A). In 1p/19q co-deleted group, 89.6% (172/192) 1p/19q co-deleted patients had a lower expression of HDAC1 than DRG2, while 100% (500/500) 1p/19q intact patients had a lower expression of DRG2 than HDAC1 (Figure 1B, p=0.0063, chi-square test). And the Receiver Operating Characteristic (ROC) curve showed that this model achieved an area under the curve (AUC) of 0.9878 (Figure 1D). With this prediction model, in the validation dataset, we got a similar result (Figure 1C, p<0.0001, chi-square test) and the AUC was 0.9165 (Figure 1E). Previously studies showed that HDAC1, locating at chromosome 1p, might serve as a good diagnostic and prognostic marker for lung cancer [26]. Overexpressing DRG2, locating at chromosome 17p, could delay cell-cycle arrest and apoptosis [27].

**Figure 2:**
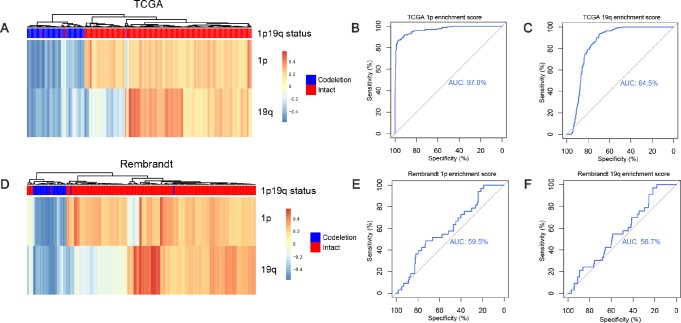
**Predicting 1p/19q status by GSVA algorithm.** (**A** and **D**) the hierarchical clustering of TCGA and Rembrandt cohorts based on the enrichment scores of 1p and 19q genes, respectively. (**B**, **E**) ROC for 1p/19q status prediction by 1p genes enrichment scores, AUC (TCGA cohort) = 0.970, AUC (Rembrandt cohort) = 0.595. (**C**, **F**) ROC for 1p/19q status prediction by 19q genes enrichment scores, AUC (TCGA cohort) = 0.845. AUC (Rembrandt cohort) = 0.567.

GSVA method was designed for integrating genes that shared common biological functions or chromosomal locations and was widely used in cancer research [24, 28, 29]. Gene set enrichment score of genes on 1p and genes on 19q were calculated with *gsva* function, respectively. Hierarchical clustering was performed based on the enrichment scores in the training dataset. Two branches were identified: 95.5% (169/177) 1p/19q co-deleted samples were clustered into the first branch while 99.6% (513/515) 1p/19q intact samples were clustered into the second branch (Figure 2A). The ROC curves of the enrichment score of 1p and 19q showed an AUC of 0.97 (Figure 2B) and 0.845 (Figure 2C), respectively. In the validation dataset, two branches were identified: 81.6% (31/38) 1p/19q co-deleted samples were clustered into the first branch, while 98.9% (182/184) 1p/19q intact samples were clustered into the second branch (Figure 2D). In the validation dataset, ROC curves of the enrichment score of 1p and 19q showed an AUC of 0.595 (Figure 2E) and 0.567 (Figure 2F), respectively.

**Figure 3:**
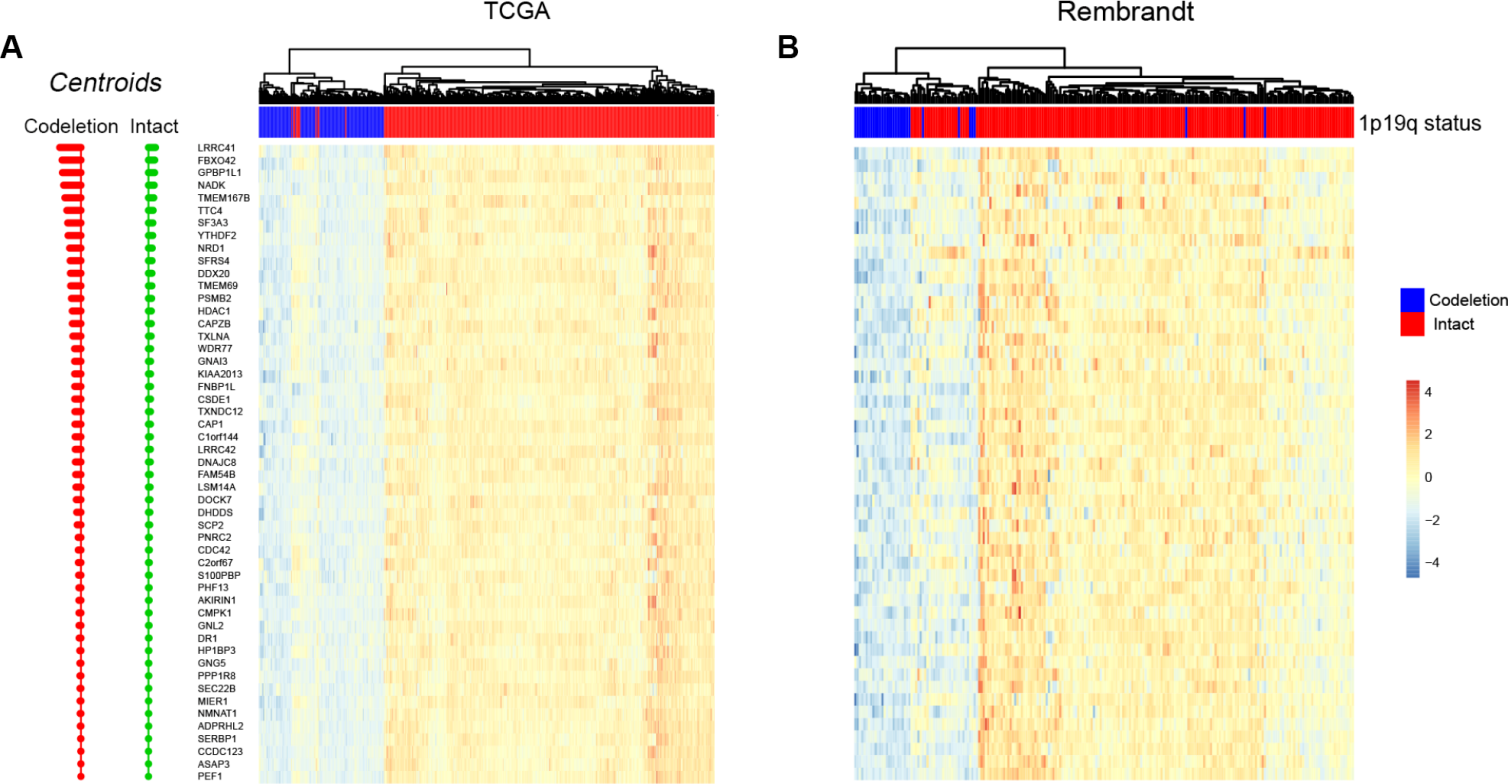
**Predicting 1p/19q status by PAM algorithm.** The hierarchical clustering samples in TCGA and Rembrandt cohorts using 53 signature genes, respectively.

PAM method exhibited powerful predictive capabilities in rectal cancer by selecting a group of genes [30]. The centroid shrinkage value of 10.984 which containing a minimum of 53 genes and 7 misclassification errors was selected in the training dataset (Figure 3A). Then the RNA expression profile of the 53 genes in the two datasets were clustered. In the training dataset, two branches were identified: the first branch contained 91.0% (172/189) 1p/19q co-deleted samples, while the second branch contained 100% (503/503) 1p/19q intact patients (Figure 3A). In the validation cohort, two branches were identified as expected. The first branch contained 45.5% (25/55) 1p/19q co-deleted patients. Meanwhile, 98.8% (164/167) 1p/19q intact patients were grouped into the second branch (Figure 3B).

**Figure 4:**
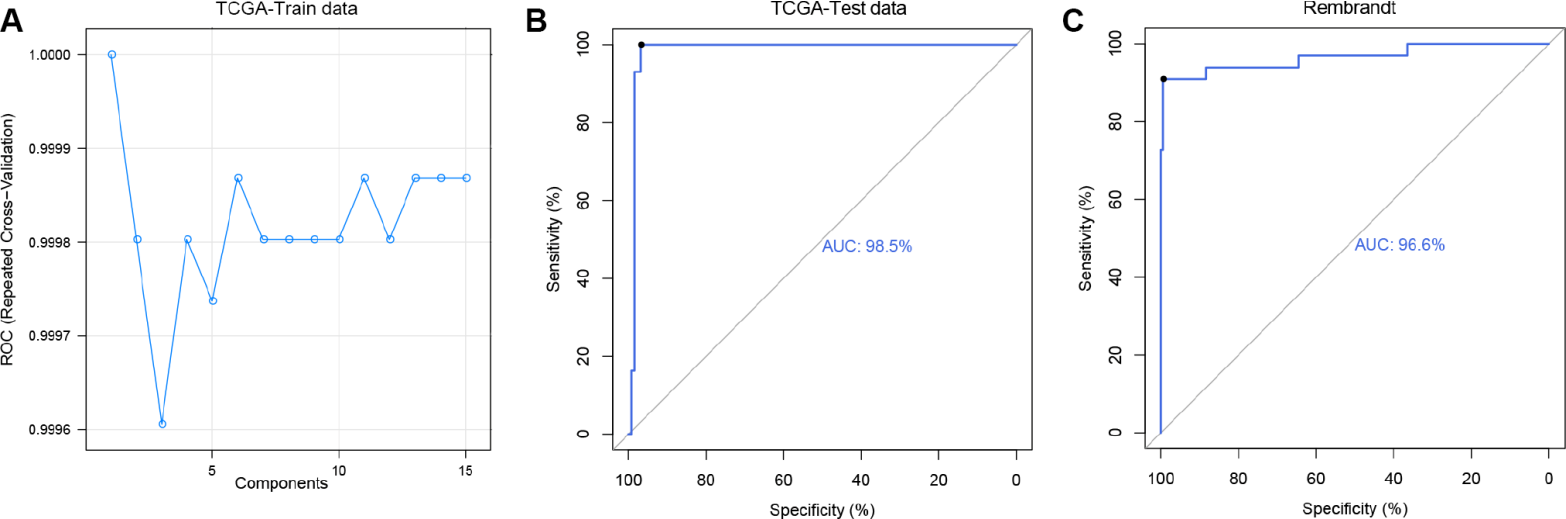
**Predicting 1p/19q status by Caret algorithm.** (**A**) The ROC values of 15 PLS models were compared to select the optimal prediction model (ncomp.1) using TCGA-Test data. (**B** and **C**) ROC curves for 1p/19q status prediction by applying the ncomp.1 model, AUC (TCGA-Test data) = 0.985, AUC (Rembrandt) = 0.966.

Caret method, a powerful machine learning algorithm, was designed for predictive modeling in practice with gene expression data and has been applied in predicting clinical outcome of patients with Alzheimer's disease [31]. In each cross validation, 20% samples in the training dataset were selected as TCGA-Train data to build predictive model and the rest of samples in the training dataset were grouped into TCGA-Test data to estimate the efficiency of the model. In the TCGA-Train dataset, the partial least squares discriminant analysis (PLSDA) method [32] was performed to build regression models and 10-fold cross-validation was used to examine the predictive efficiency [33]. As shown in ROC curve, the AUC was more than 0.999 in every repeated cross-validation cohort (Figure 4A) and the maximum value was 1.0 (ncomp=1). Then we predicted 1p/19q status with the best model. In the TCGA-Test dataset, 93.48% (43/46) 1p/19q co-deleted patients and 100% (126/126)1p/19q intact patients were successfully predicted. And in the validation dataset, 85.7% (30/35) 1p/19q co-deleted patients and 98.4% (184/187) 1p/19q intact patients were successfully predicted. The AUC values for TCGA-Test dataset and validation dataset were 0.985 (Figure 4B) and 0.966 (Figure 4C), respectively.

**Figure 5:**
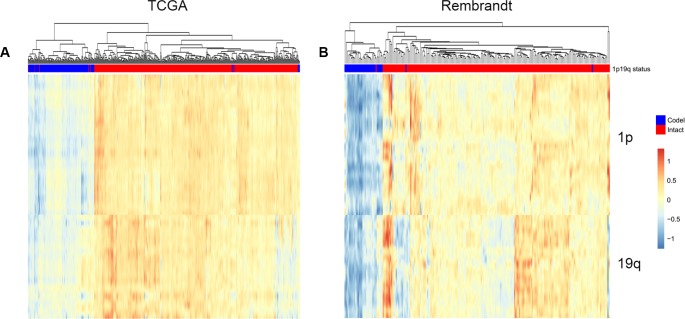
**Predicting 1p/19q status by smoother algorithm.** The hierarchical clustering of TCGA and Rembrandt cohorts by using smoothed gene expression on 1p and19q respectively.

Smoother method, which could modify individual noise, have been performed in the analysis of esophageal squamous cell carcinoma [34] and breast cancer [35]. Firstly, genes expression data in 1p and 19q from start to end were smoothed by a 100 genes window. Then the combined 1p and 19q smoothed RNA expression profile were clustered. In the training dataset, two branches were clustered: 96.4% (163/169) 1p/19q co-deleted patients were clustered into the first branch, while 98.3% (514/523) 1p/19q intact patients were clustered into the second branch (Figure 5A). In the validation dataset, 96.9% (31/32) 1p/19q co-deleted patients were in the first branch, while 98.9% (188/190) 1p/19q intact patients were in the second branch (Figure 5B).

### Comparing the prediction accuracy of five methods

**Figure 6:**
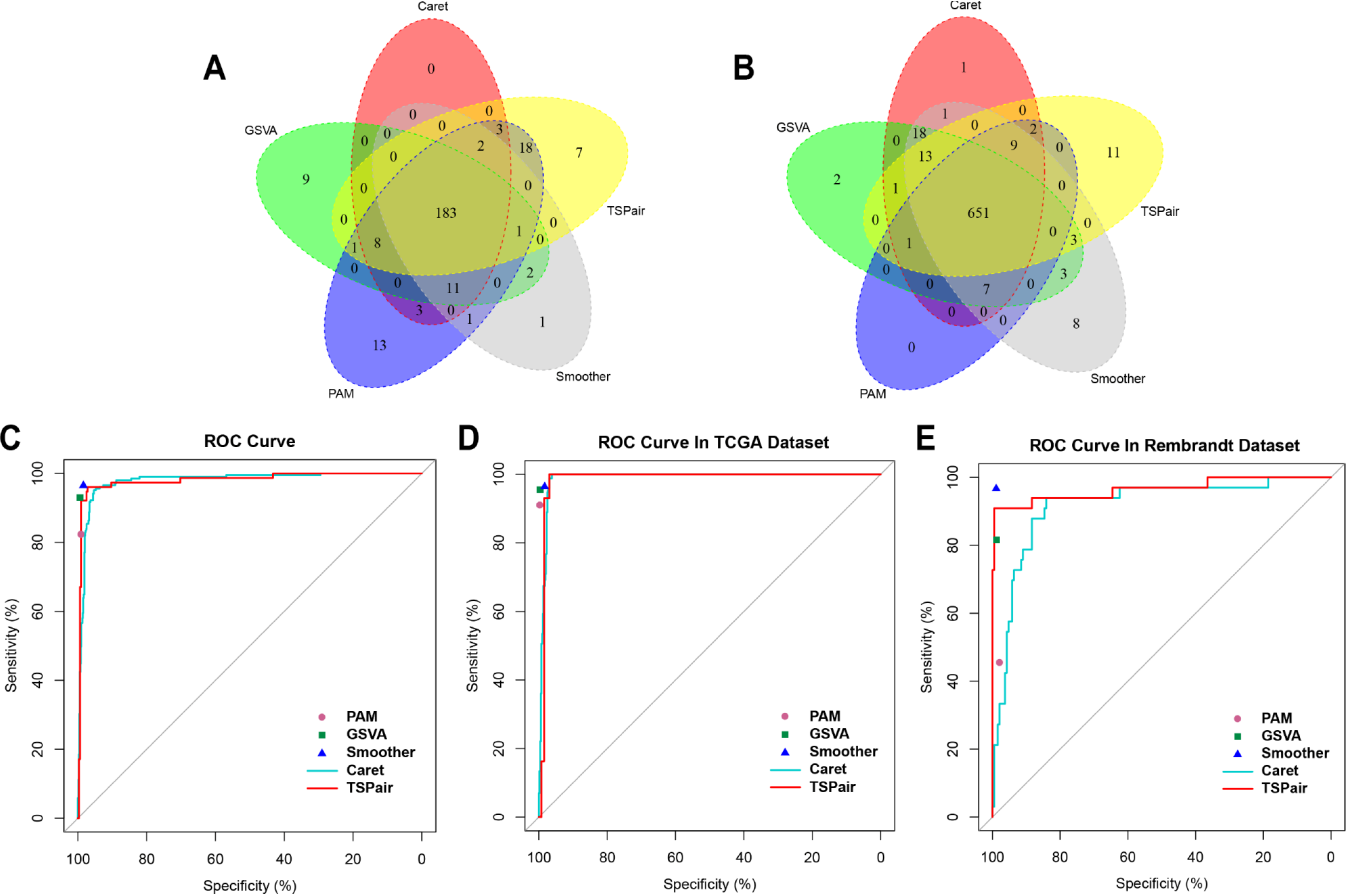
**Comparing the sensitivity, specificity and accuracy of five algorithms.** (**A**) Overlap among of the 1p/19q co-deleted samples found by five methods. (**B**) Overlap among of the 1p/19q intact samples found by five methods. (**C**) Comparing the sensitivity and specificity of five algorithms in predicting 1p/19q status in 914 samples. (**D**) Comparing the sensitivity and specificity of five algorithms in predicting 1p/19q codeletion in TCGA dataset. (**E**) Comparing the sensitivity and specificity of five algorithms in predicting 1p/19q codeletion in Rembrandt dataset.

Finally, we summarized the similarities and differences between five methods and evaluated the most appropriate method to predict 1p/19q status with ROC curves. The 1p/19q status results that identified by single nucleotide polymorphism (SNP) array were used as golden standard in our study. As shown in the Figure 6A a total of 183 patients were classified into 1p/19q co-deleted group by five RNA processing methods and 181(88.3%) of them were identified by SNP array. While 99.7% (649/651) 1p/19q intact patients were identified by SNP array (Figure 6B).

Meanwhile, when integrated samples in TCGA and Rembrandt dataset, the smoother (96.5%) and PAM (82.3%) exhibited the highest sensitivity and lowest level of sensitivity among five methods, respectively (Figure 6C). And TSPair (97.9%) showed the lowest level of specificity. The sensitivity and specificity of five methods in predicting 1p/19q status in TCGA dataset exhibited similar trends (Figure 6D). Except TSPair (89.6%), the sensitivities of other methods were all more than 90% and smoother (96.5%) was still the highest one. The TSPair (100%) and PAM (100%) methods had the highest specificity while smoother method (98.3%) had the lowest specificity. However, in Rembrandt dataset, the sensitivities of GSVA (81.6%), PAM (45.5%), Caret (85.7%) and TSPair (64.5%) were all decreased obviously. Only smoother (sensitivity = 96.7%, specificity = 98.8%) method remained a higher accuracy in predicting the 1p/19q status.

After synthetical comparing the differences of sensitivity, specificity and accuracy among five methods in two individual datasets, we found that smoother method clearly outperformed the other methods in predicting 1p/19q status with little room for improvement.

## DISCUSSION

With the rapid expansion of multi-platform integrated analysis of glioma, molecular markers have greatly facilitated the understanding of the genetic progress underlying the progress of cancer and provided key insights on precision medicine. The status of chromosome 1p/19q is one of the most crucial molecular markers in glioma, which has shown well-established association with the diagnosis and prognosis of patients [36].

DNA sequencing, processing the order of nucleotides in DNA, is considered as the most accurate method in detecting large regions chromosome variation [37]. However, the cost to generate a high-quality DNA sequencing was almost $2,500 and the medical insurance does not cover the cost for genetic tests [38]. DNA sequencing as routine tool to detect 1p/19q status was limited due to the high cost. There is some uncertainly about detecting 1p/19q status with FISH method due to the limitations. Thus, quality assurance remains an issue, finding a more cost-effective and accurate way to obtain 1p/19q status information would greatly promote the widespread of genetic-guided precision medicine of glioma. Next-generation sequencing technologies have revolutionarily advanced genome-related research with the advantages of high-throughput, high-sensitivity, and low-cost [39]. RNA-seq is now being used widely for detecting molecular aberrations in cancer research [40]. It is well known that the copy number variations (CNVs) typically result in a corresponding gene expression changes, especially large chromosome amplification or deletion [41]. CNV-related gene expression changes supported us detecting 1p/19q status with RNA sequencing data.

The main purpose of this study was to explore an appropriate RNA sequencing computational method to detect 1p/19q status. In this study, several analysis pipelines have been applied to analyze gene expression data for predicting 1p/19q status. TSPairs method, which identifies an alteration by comparing ratio of two genes expression, has been used in breast cancer and lung adenocarcinoma for molecular classification and predicting prognosis [24, 25]. GSVA method is designed for interpreting gene expression data. By using GSVA method, scientists are able to get further insights into leukemia and lung cancer [42, 43]. PAM method exhibits powerful predictive capabilities in rectal cancer by selecting a group of genes [30]. Caret method relates the practice of predictive modeling and has been applied in predicting prognosis of Alzheimer's disease [31]. Smoother method is designed for removing the noise and scatter of RNA sequencing data [44]. Finally, smoother method was selected as the most stable and accurate method in TCGA and Rembrandt datasets.

There were some advantages in inferring 1p/19q status with gene expression data. Firstly, the cost in RNA sequencing is much less than FISH. Prediction 1p/19q status with RNA sequencing data could reduce financial burden for patients. Secondly, identifying 1p/19q status by gene expression data could eliminate the limitation of FISH probes of testing only two regions. Thirdly, the RNA sequencing data could also be applied to call or predict IDH mutation, ATRX mutation, TERT mutation, EGFRvIII deletion, fusion genes and so on. The integrated analysis of DNA sequencing technology, RNA sequencing technology and FISH were shown in Supplementary Table 1. The experiment in RNA sequencing used the shortest time and least amount of money.

However, several limitations should be considered in this research. Firstly, the prediction models based on five RNA sequencing data processing methods were established retrospectively. The prospective longitudinal study was need to estimate this research in future. Second, the consistency between FISH and prediction models in predicting 1p/19q status could not be evaluated due to the limitation of FISH information of glioma patients in TCGA database and REMBRANDT database.

In summary, along with the application of next-generation sequencing in clinical practice, we believed that RNA sequencing processing method will show great potential as the standard detection method to detect various genetic and molecular alterations. For detecting 1p/19q, we would recommend smoother method using RNA sequencing data, which was more cost-effective and convenient in clinical practice.

## MATERIALS AND METHODS

### Data collection

Training set: RNA-sequencing data of 692 glioma samples downloaded from The Cancer Genome Atlas (TCGA, http://cancergenome.nih.gov/), containing 172 1p/19q co-deleted and 520 1p/19q intact patients. TCGA RNA-seq expression data was log2 transformed before using. Validation set: RNA microarray expression data of 222 glioma samples from Repository for Molecular Brain Neoplasia Data (Georgetown Data-base of Cancer G-DOC, https://gdoc.georgetown.edu/gdoc/), containing 33 1p/19q co-deleted and 189 1p/19q intact patients. The characteristics of glioma patients were described in Table 1.

**Table 1:** Clinicopathological characteristics of the patients

Variable		TCGA dataset	Rembrandt dataset
Age	≥45	333	69
	<45	296	103
	NA	63	50
Gender	Male	364	124
	Female	265	56
	NA	63	42
Preoperative KPS score	≥80	320	-
	<80	70	-
	NA	302	222
Grade	II	223	27
	III	245	29
	IV	161	131
	NA	63	35
IDH1/2 status	Mutation	440	-
	Wild type	242	-
	NA	10	222
1p/19q status	Codeleted	172	33
	Intact	520	189
Molecular subtype	Astrocytoma (II, III)	170	50
	Oligoastrocytoma (II, III)	118	6
	Oligodendroglioma (II, III)	180	35
	Glioblastoma	161	131
	NA	63	0

### Running predictors

We evaluated the efficacy of five different methods for identifying 1p/19q status in two datasets. These methods were described as bellow:

*Top scoring pairs (TSPairs)* contains functions for selecting top scoring pairs whose relative rankings can be used to accurately classify individuals into one of two classes (20501*20500 =420500270 gene pairs) [45].

*Gene Set Variation Analysis (GSVA)* is a non-parametric, unsupervised method for estimating variation of gene set enrichment through the samples of an expression data set and bypasses the conventional approach of explicitly modeling phenotypes within the enrichment scoring algorithm [28].

*Prediction analysis for microarrays (PAM)*, which can be defined as a ‘nearest shrunken centroid classifier’ is a statistical method for class prediction by adding a ‘fudge-factor’ to each statistic's denominator [46]. TCGA and Rembrandt datasets were removed batch effects before using pam method.

*Functions Relating to the Smoothing of Numerical Data (smoother)* could smooth numerical data, blur images and remove detail and noise. The gaussian window smoothing function allows users to infer the pattern of DNA aberration from gene expression [47].

*Classification and Regression Training(caret)*, a machine learning algorithm, could integrate with the features in training and the modeled interaction features. The model was evaluated independently through stratified (k=10)-folds cross-validation [48].

Hierarchical clustering was performed using complete agglomeration algorithm and a distance metric equal correlation coefficient with data processed by PAM, smoother and GSVA methods. We obtained TSPairs method from R package “tspair”, GSVA method from R package “GSVA”, PAM method from R package “pamr”, smoother method from R package “smoother”, caret method from R package “caret”, Combat function for removing batch effects [49] among TCGA and Rembrandt datasets from R package “sva” and hierarchically clustered function from R package “pheatmap”. All packages were based on the statistical software environment R, version 3.3.4 (http://www.r-project.org).

## SUPPLEMENTARY MATERIAL

Supplementary Figure

Supplementary Table
