# Supplementary material for: Predicting chromosome 1p/19q codeletion by RNA expression profile: a comparison of current prediction models
Source: Aging (Albany NY). 2019 Feb 2;11(3):974–85. doi: 10.18632/aging.101795 (PMC6382420; doi:10.18632/aging.101795)
Supplement: Supplementary Figure [file aging-11-101795-s001.pdf]

SUPPLEMENTARY FIGURE

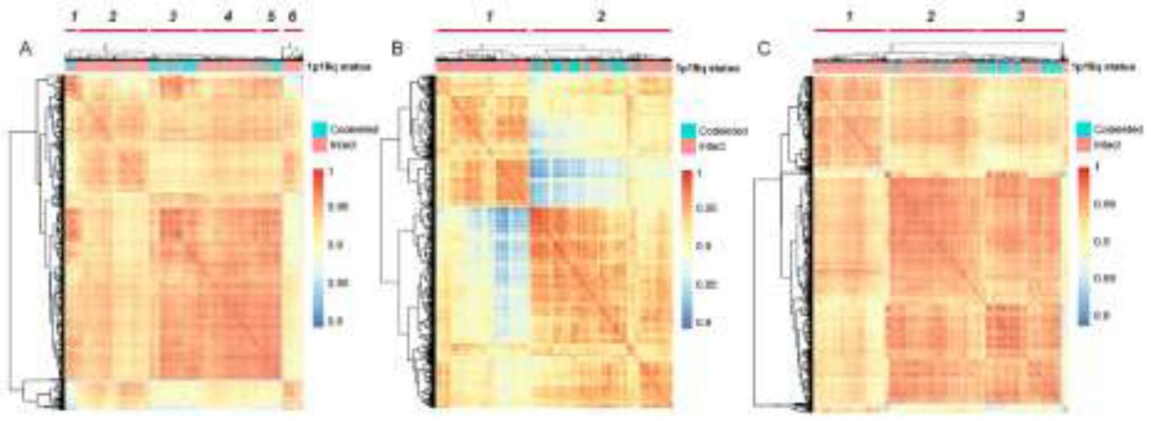

Supplementary Figure 1. Expression profile revealed a distinct gene expression between Chromosome 1p19q codeleted and intact patients. (**A**) Hierarchical clustering based on whole genome expression profiling (20501 genes), there were 92.4% (159/172) 1p/19q co-deleted patients in group1, 3 and 5 and 85.7% (446/520) 1p/19q intact patients in the rest of groups. (**B**) Hierarchical clustering based on highly variable expression genes (MAD >2, 886 genes), there were 96.5% (166/172) 1p/19q co-deleted patients in group2. (**C**) Hierarchical clustering based on genes on chromosome 1p and 19q (1775 genes), there were 75% (129/172) 1p/19q co-deleted patients in group3
