# Supplementary material for: Predicting chromosome 1p/19q codeletion by RNA expression profile: a comparison of current prediction models
Source: Aging (Albany NY). 2019 Feb 2;11(3):974–85. doi: 10.18632/aging.101795 (PMC6382420; doi:10.18632/aging.101795)
Supplement: Supplementary Table [file aging-11-101795-s002.pdf]

SUPPLEMENTARY TABLE

Supplementary Table 1. Integrated analysis of methods for detecting 1p/19q status.

Methods	Cost	Time	Accuracy
Whole genome sequencing	~$2500	~1month	100%
RNA sequencing	~$200	~2weeks	97.8%
FISH	~$340	~2weeks	1p36/1q21 and 19q13/19p13 Deletion Probe Kit
